# RAPID-SELEX for RNA Aptamers

**DOI:** 10.1371/journal.pone.0082667

**Published:** 2013-12-20

**Authors:** Kylan Szeto, David R. Latulippe, Abdullah Ozer, John M. Pagano, Brian S. White, David Shalloway, John T. Lis, Harold G. Craighead

**Affiliations:** 1 School of Applied and Engineering Physics, Cornell University, Ithaca, New York, United States of America; 2 Department of Molecular Biology and Genetics, Cornell University, Ithaca, New York, United States of America; Deutsches Krebsforschungszentrum, Germany

## Abstract

Aptamers are high-affinity ligands selected from DNA or RNA libraries via SELEX, a repetitive in vitro process of sequential selection and amplification steps. RNA SELEX is more complicated than DNA SELEX because of the additional transcription and reverse transcription steps. Here, we report a new selection scheme, RAPID-SELEX (RNA Aptamer Isolation via Dual-cycles SELEX), that simplifies this process by systematically skipping unnecessary amplification steps. Using affinity microcolumns, we were able to complete a multiplex selection for protein targets, CHK2 and UBLCP1, in a third of the time required for analogous selections using a conventional SELEX approach. High-throughput sequencing of the enriched pools from both RAPID and SELEX revealed many identical candidate aptamers from the starting pool of 5×10^15^ sequences. For CHK2, the same sequence was preferentially enriched in both selections as the top candidate and was found to bind to its respective target. These results demonstrate the efficiency and, most importantly, the robustness of our selection scheme. RAPID provides a generalized approach that can be used with any selection technology to accelerate the rate of aptamer discovery, without compromising selection performance.

## Introduction

Aptamers are high-affinity ligands selected from large libraries of random oligonucleotides that can contain up to 10^16^ unique sequences. SELEX (Systematic Evolution of Ligands by EXponential enrichment) [1]–[3], an in vitro selection method, can isolate aptamers with high-affinity and specificity for a wide range of target molecules from DNA or RNA libraries [4]–[6]. This is achieved by iteratively selecting and amplifying target-bound sequences to preferentially enrich those sequences with the highest affinity to the target. Traditionally, after 10 to 15 iterations, one or several aptamers may be identified from the enriched pool, a process that may take months to complete. If an RNA aptamer is desired, this process takes even longer due to additional steps required for reverse transcription to amplifiable cDNA and subsequent transcription back to RNA. A disproportionate amount of time and effort is dedicated to amplifying RNA pools compared to the actual selection steps where aptamer enrichment takes place.

Recent work has focused on improving selection efficiency and enriching for aptamers with particular target-binding properties. This has resulted in modifications to the conventional SELEX strategy including the use of multiple targets to control specificity [7]–[9], changing the characteristics of the nucleic acid library [10]–[16], using different substrates for presentation of target molecules [1], [17]–[20], and varying the separation technique [1], [17], [21], [22]. Work has also been done to improve the throughput of aptamer discovery by utilizing high-throughput sequencing [17], [23]–[26] or by performing parallel selections [19], [27]. A number of automated selection strategies have also been introduced [28]. However, fully automated systems lack the quality controls and evaluations that are applied when manual selections are performed [29]. Recently, we reported a multiplexed microcolumn technique that optimized selection parameters based on enrichment of a specific aptamer and demonstrated the ability to efficiently perform selections against multiple targets in parallel [30]. However, there is still a lack of thorough characterization and knowledge about the most efficient or effective methods and conditions for performing selections with emerging technologies. Improvements in this domain would not only increase the rate of aptamer selections, but have the potential to improve the rate and quality of downstream aptamer identification and refinement [30], [31].

Despite many advances, only a few selection approaches diverge from the core methodology of traditional SELEX. To our knowledge, only one method breaks from the typical cycle of iterative and sequential selection and amplification steps; Non-SELEX [32] was shown to quickly generate DNA aptamers by repeated selections from an enriched library without any amplification steps. This methodology only takes about an hour to complete and is particularly useful for libraries that cannot be amplified. However, the capillary electrophoresis-based platform used for Non-SELEX requires tiny injection volumes (∼150 nL) to achieve efficient separations and only a small fraction of the sequences recovered from a given selection cycle are re-injected for the subsequent cycle. This constraint significantly lowers the total number of sequence candidates that can be investigated, decreasing the complexity and diversity of the injected library by 5 or 6 orders of magnitude. Despite these restrictions, Non-SELEX was successfully used to identify DNA aptamers to h-RAS protein, bovine catalase and signal transduction proteins [32]–[34], which suggests that in some cases aptamers may be much more abundant in random pools than previously thought. However, without the amplification steps utilized in traditional SELEX, this technique makes identifying aptamer candidates via population-based methods difficult. This limits the potential for using high-throughput sequencing, which has been used to characterize sequence distributions and their cycle-to-cycle dynamics, and has proven to be a powerful technique for identifying enriching aptamers with great sensitivity [17], [23], [25], [26], [30].

Here we propose a new scheme, RNA Aptamer Isolation via Dual-cycles SELEX (RAPID-SELEX or RAPID for short), which combines the efficiency of Non-SELEX with the robustness of conventional SELEX and provides a generalized approach for accelerating the rate of aptamer selections. RAPID significantly decreases the time required for RNA aptamer selections by systematically eliminating unnecessary amplification steps and performing amplifications only when higher numbers of certain sequences (referred to as the copy number) or higher pool concentrations are required. This results in a process that maximizes enrichment per unit time, rather than enrichment per cycle. For each additional selection cycle performed without amplification (Non-Amplification Cycle), the additional effort associated with RNA specific processes, such as reverse transcription and transcription is eliminated in addition to the typical PCR amplification of DNA templates. Furthermore, RAPID can be applied to any selection mode and used with any technology, including those that utilize whole cells and target cell surface proteins as in Cell-SELEX [18]. We demonstrate the improved efficiency of RAPID, by comparing and analyzing its sequence candidates to those generated from conventional SELEX using our previously described, microcolumn-based platform [30] to the target proteins, CHK2 and UBLCP1. CHK2 and UBLCP1 are a kinase and a phosphatase, respectively, and were chosen because they were readily available and no aptamer selections had been previously performed against them. After completing six selection cycles, RAPID had enriched many of the same candidates, but in only a third of the time required for conventional SELEX.

## Results

### SELEX versus RAPID

**Figure 1:**
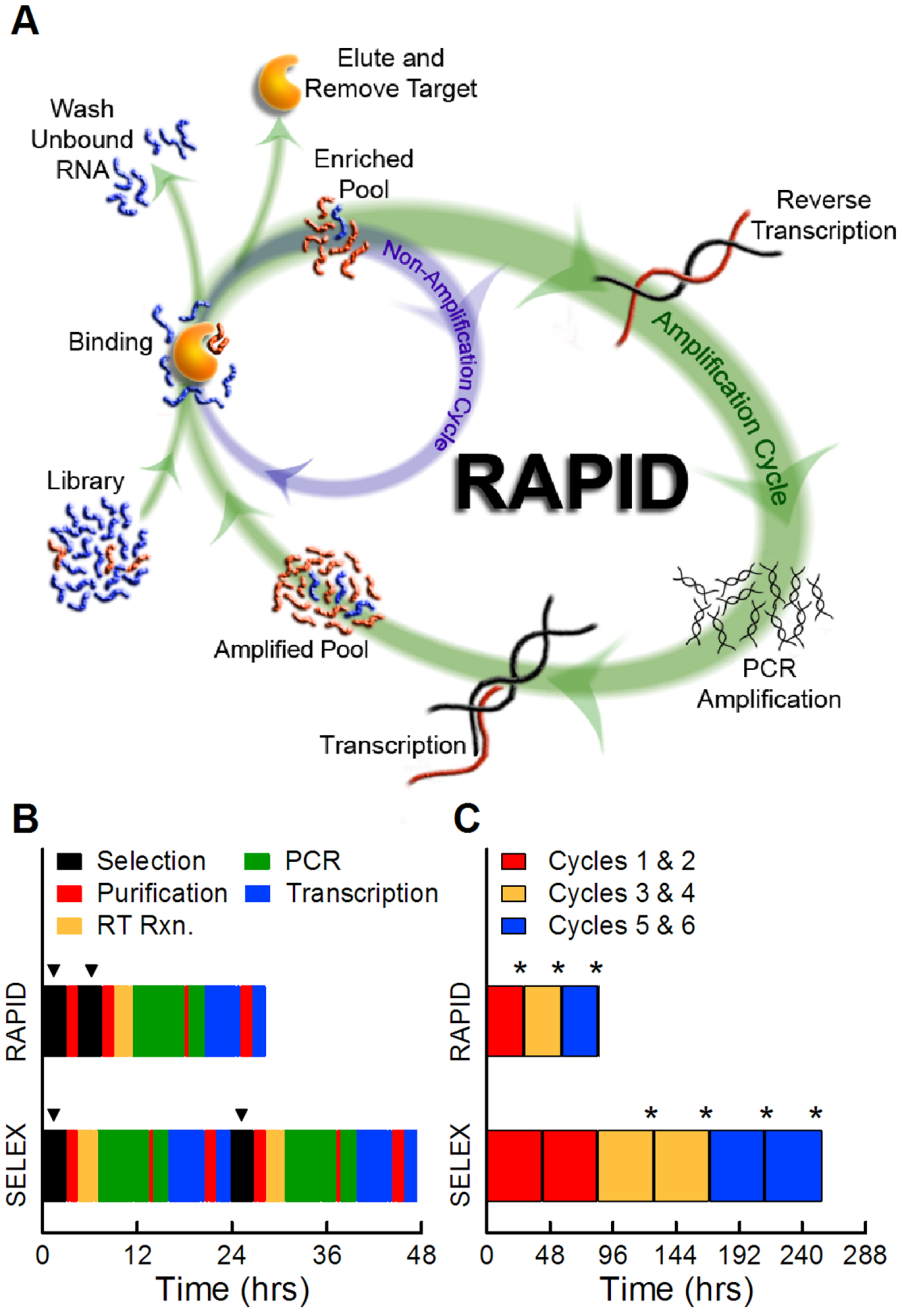
RNA Aptamer Isolation via Dual-cycles (RAPID). (A) Diagram of the RAPID process. The starting library or the enriched pool from the previous selection step can either go through the (inner) Non-Amplification Cycle and be used immediately in the next selection or go through the regular (outer) Amplification Cycle. (B) An example of processing times for SELEX and RAPID to complete two full selection cycles. Each selection is indicated with black blocks and arrowheads (▾) on top. (C) The total time required to complete six cycles of SELEX under optimal enrichment conditions, and six cycles of RAPID performed by alternating between Non-Amplification and Amplification Cycles; each colored block represents the total processing time between amplification steps. Asterisks (*) indicate the enriched and amplified pools that were analyzed via high-throughput sequencing.

Traditional SELEX is performed with a random library via iterative cycles of sequential steps (binding, partitioning, and amplification of target-bound sequences) until an aptamer emerges. To improve the efficiency of these selections, we developed and tested a hybrid selection scheme between SELEX and Non-SELEX that utilizes two cycles; one that includes amplifications and one which does not. For simplicity, we differentiate these two cycles as Amplification and Non-Amplification Cycles (Figure 1A). By systematically eliminating certain amplification steps, RNA selections can be performed in much less time, and require less reagents and other costly materials. In addition, removing unnecessary amplification steps minimizes their potential biases [24], [35] and also reduces large input libraries and pools to more convenient size scales when performing amplifications. Thus, rapid sequence convergence can be obtained via Non-Amplification Cycles, while diverse sequence populations with high aptamer copy numbers are maintained through critical periodic Amplification Cycles.

To illustrate the validity of the RAPID method for RNA aptamer selections, we compared the simplest RAPID protocol (a single non-amplification cycle followed by an amplification cycle) to conventional SELEX (amplification at every cycle). Representative timelines for two cycles of RAPID and conventional SELEX conducted with the exact same selection conditions are shown in Figure 1B. Completion of one cycle of conventional SELEX takes about 24 hours, over 80% of which is needed for the amplification step. In contrast, by adding one Non-Amplification Cycle, RAPID completes two selection cycles in nearly the same amount of time. For both methods, we define a selection “round” to necessarily include the amplification steps. In this way, a round of RAPID is comparable in time and effort to a round of SELEX; a round and a cycle are interchangeable terms in conventional SELEX.

To evaluate the advantage of using RAPID, we completed six selection cycles on the same set of targets using both the RAPID and conventional SELEX methods. As shown in Figure 1C, SELEX took a total of 255 hours using the optimal parameters for aptamer enrichment on the microcolumns as determined in our previous work [30]. RAPID took only 84 hours to complete the three rounds with six selection cycles (Figure 1C). However, different parameters were used to allow for the completion of two selection steps within one working day (i.e. a 10 hour time period). With this simple design, RAPID was straightforward to execute and took one third the time to complete as SELEX. If the same selection step parameters were used for both processes, RAPID would have been completed in half the time needed for SELEX (Figure 1B).

### Ensemble Binding of Enriched Aptamer Pools

**Figure 2:**
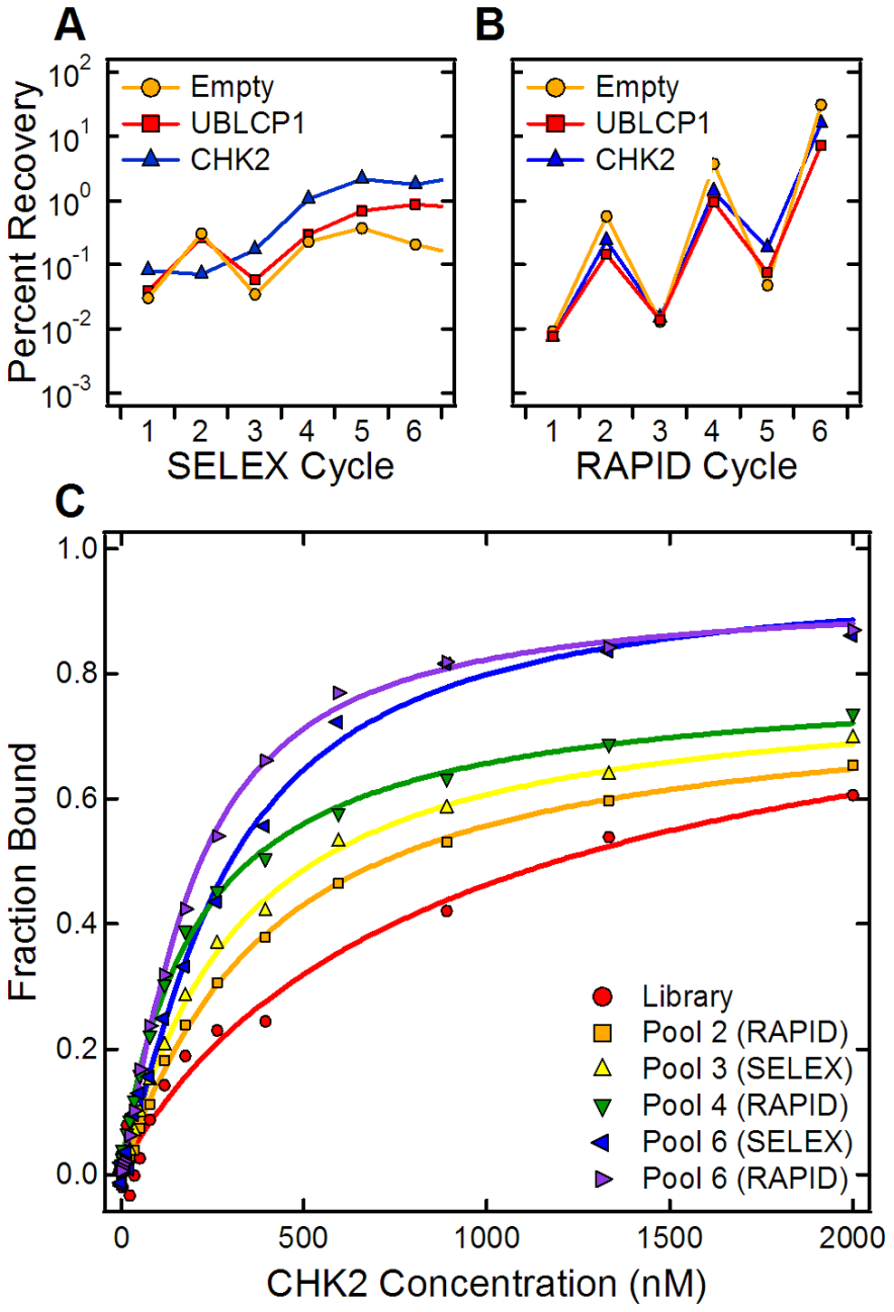
Binding of RNA after each selection cycle. (A) Percent RNA recovery for SELEX cycles for Empty (orange circles), UBLCP1 (red squares), and CHK2 (blue triangles) microcolumns. In this mode, there is a clear distinction between the protein-bound and the Empty microcolumns. (B) Percent RNA recovery for RAPID cycles for the same targets. In this mode, there are significant increases in the percent aptamer recoveries following selections with non-amplified pools at Cycles 2, 4, and 6, followed by a concentration induced drop with the amplified pools at Cycles 3 and 5. (C) Test of enriched pool binding to CHK2 protein preparation. F-EMSA shows the progression of bulk binding affinity increase for both SELEX and RAPID enriched pools with the RAPID Cycle 6 pool showing higher bulk binding than the SELEX Cycle 6 pool.

To monitor the progress of the selections, the recovery of bound RNA during each selection step was measured using quantitative PCR (qPCR). Figure 2A shows the results for all six SELEX cycles to the Empty, UBLCP1 and CHK2 microcolumns. An increase in the fraction of bound RNA was observed from cycle to cycle for all three samples. The empty microcolumns generally bound an amount of RNA comparable to that bound to the microcolumns containing the two protein targets. This is because nearly all the recovered sequences in early selection cycles represent background and non-specific binding sequences. However, the two protein targets show higher recoveries than the Empty microcolumn, with the CHK2 target demonstrating the highest levels for the later cycles. Figure 2B shows the results for all six cycles of RAPID to the same three targets. The recovery of the aptamer library with the RAPID method showed fluctuations from cycle to cycle that we believe are characteristic of the varying input concentrations since the total amount of material available following a Non-Amplification Cycle (1, 3, and 5) is lower compared to that following an Amplification cycle. This effect causes an increase in the recovery observed during the Amplification cycle. Despite these concentration induced fluctuations, CHK2 consistently showed the higher recovery of the two protein targets.

To evaluate improvements in target binding, Fluorescence Electrophoretic Mobility Shift Assays (F-EMSA) were performed with the initial random library and five enriched pools from the selection cycles for the CHK2 protein: RAPID cycle 2, SELEX cycle 3, RAPID cycle 4, SELEX cycle 6, and RAPID cycle 6. For each pool, the percent of input RNA that was bound at the highest protein concentration and the apparent ensemble dissociation constant, K_d-app_, were calculated. The latter was determined by fitting the F-EMSA data to the Hill equation. The results shown in Figure 2C indicate a general improvement in bulk affinity and an increased pool binding fraction at later cycles. The input library had a K_d-app_ value greater than 1 µM, with 59% of input RNA bound. For SELEX, the Cycle 3 pool had a K_d-app_ = 315±26 nM (69% bound) while the Cycle 6 pool had K_d-app_ = 281±24 nM (86% bound). For RAPID, the Cycle 2, 4, and 6 pools had K_d-app_ values of 390±34 nM (65% bound), 209±19 nM (72% bound), and 191±7 nM (87% bound), respectively. Across the cycles, the fraction of bound RNA increased monotonically from 59% for the starting library to 87% for the RAPID cycle 6 pool. In addition, the RAPID Cycle 6 pool showed a slightly higher bulk affinity for the protein than the SELEX Cycle 6 pool, which suggests that RAPID was enriching pools comparably to SELEX.

### Population Distributions from High-throughput Sequencing Analysis of Selection Pools

**Figure 3:**
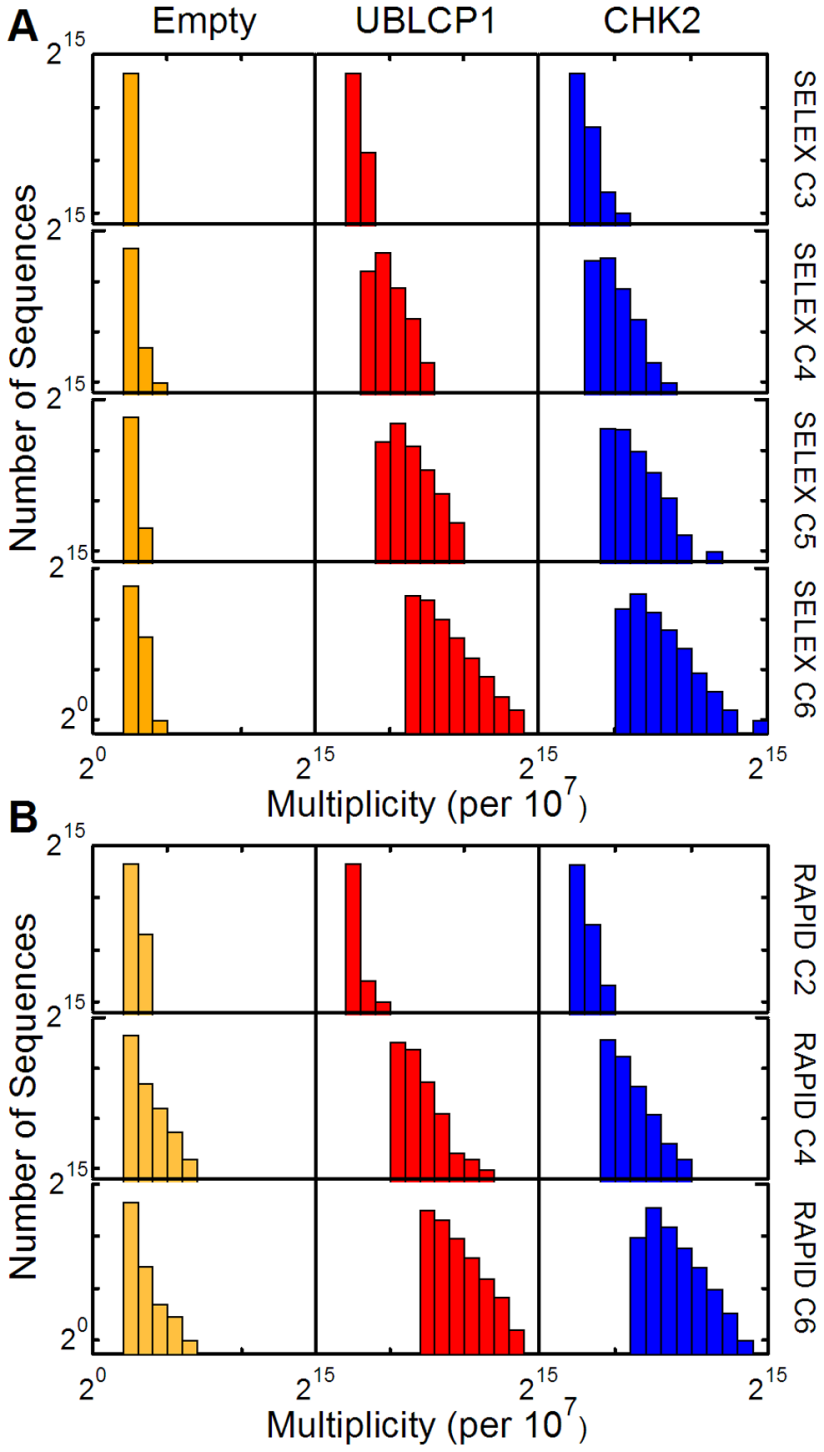
Sequence multiplicity distributions for various cycles of SELEX and RAPID. (A) Distributions of the top 10,000 Empty, UBLCP1 and CHK2 sequences for SELEX Cycles 3 to 6. (B) The same Sequence multiplicity distributions of RAPID Cycles 2, 4 and 6 for the same targets.

High-throughput sequencing was performed on selected pools to identify candidate aptamers and to compare the cycle-to-cycle enrichments of specific sequences from both the RAPID and conventional SELEX pools. As indicated in Figure 1C, the four SELEX pools for Cycles 3, 4, 5, and 6 and all three of the amplified RAPID pools were sequenced. Because the total number of sequencing reads for each pool varied between 5.6 and 9.4 million reads, the multiplicity of each sequence (number of times each sequence appeared) was normalized to 10^7^ reads. We chose to analyze the sequences with the highest multiplicity (top 10,000) from each pool, because this was sufficient to cover 10–20% of the total sequence reads from the Cycle 6 pools. The top 10,000 sequences for each pool are plotted as a histogram to compare the population distributions for each of the RAPID and SELEX pools in Figure 3A and 3B, respectively. The histograms clearly show the convergence of the protein targets’ sequences toward higher multiplicities at higher cycle numbers. As expected, there was minimal increase in multiplicity observed in the Empty columns which is consistent with the notion that RNA molecules bind randomly and non-specifically to the Empty column without enriching any specific RNA sequence. Overall, the two methods appear to be converging sequences at similar rates suggesting that RAPID’s Non-Amplification cycles perform comparably to SELEX cycles (a quantitative comparison shows that the RAPID pools are actually more converged than the SELEX pools; Figure S1).

### Multiplicity versus Cycle 4 to Cycle 6 Enrichments

**Figure 4:**
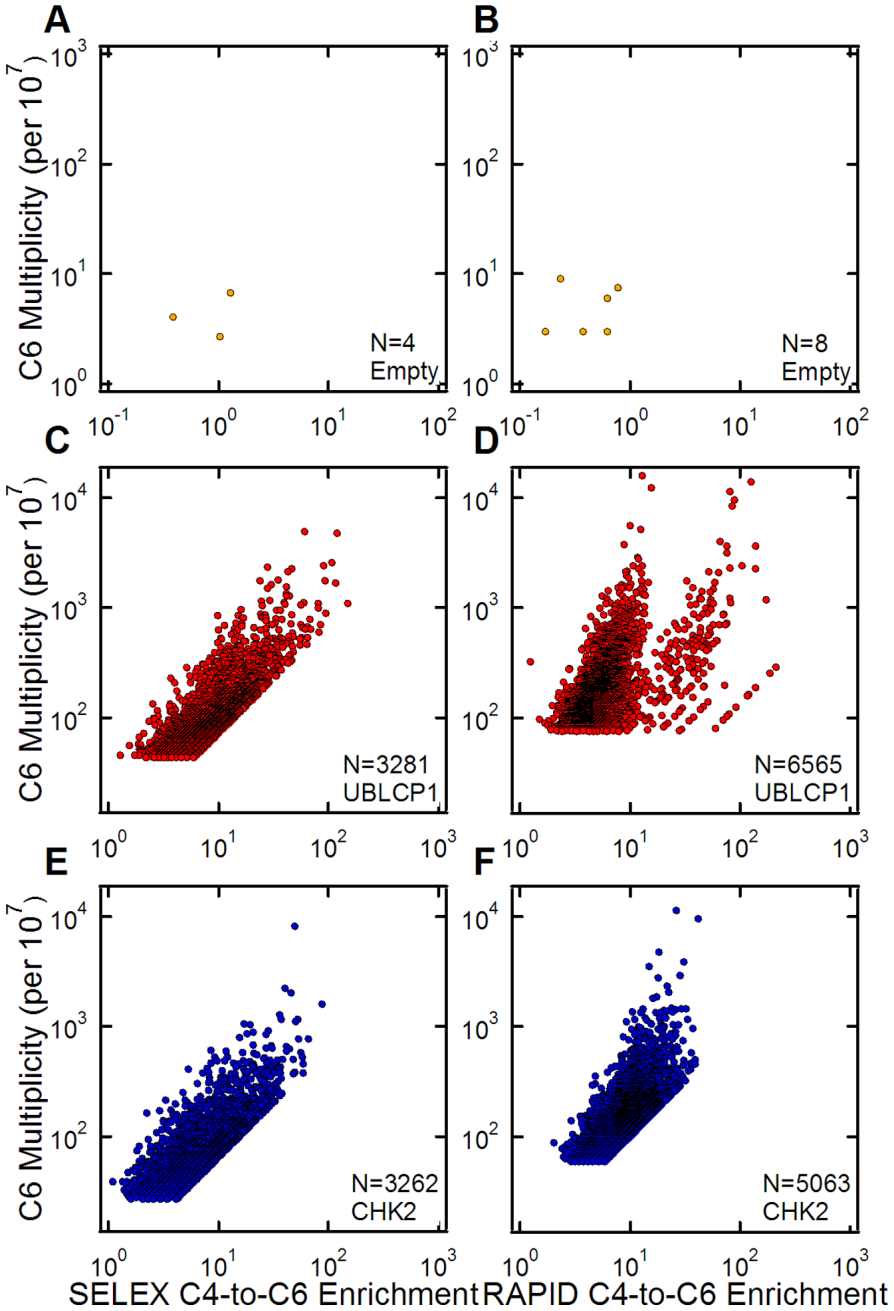
The relationship between sequence multiplicity and enrichment. (A and B) Scatter plots of sequences’ multiplicity and enrichment within the top 10,000 highest multiplicity sequences from Cycle 6 of SELEX and RAPID for the Empty microcolumns. Multiplicity values have been normalized to counts per 10^7^ and enrichment is calculated as the ratio of Cycle 6 multiplicities to Cycle 4 multiplicities for any sequence found in both pools. Some data points are obscured due to overlapping values. (C and D) Scatter plots of sequences’ multiplicity and Cycle 4-to-Cycle 6 enrichment within the top 10,000 highest multiplicity sequences from Cycle 6 of UBLCP1 SELEX and RAPID. (E and F) Scatter plots of sequences’ multiplicity and enrichment within the top 10,000 highest multiplicity sequences from Cycle 6 of CHK2 SELEX and RAPID. RAPID sequences show significantly higher multiplicities at lower enrichments than SELEX.

To further investigate and compare the evolving RNA pools obtained with RAPID and SELEX, the enrichments of individual sequences were calculated from the ratio of multiplicity values from two cycles [17]. The multiplicity values for the top 10,000 sequences in Cycle 6 were plotted versus their corresponding enrichment values from Cycle 4 for both selection methods (Figure 4). For both protein targets, these two metrics were well correlated. However, the RAPID pools (Figure 4D and 4F) have higher multiplicities at equivalent enrichments than the SELEX pools (Figure 4C and 4E), and more of the top enriched sequences were identified in Cycle 4 of RAPID. In the RAPID pools, UBLCP1 and CHK2 had 6,565 and 5,063 sequences, respectively, in common between the Cycle 4 and 6 pools’ top 10,000 sequences. For comparison, in the SELEX pools, UBLCP1 and CHK2 had 3,281 and 3,262 sequences, respectively, ranking in the top 10,000 of both pools. Thus, the RAPID pools have almost twice as many preserved sequences between cycles over SELEX, which is consistent with the improved convergence and enrichment data. In contrast, Figures 4A and 4B show that the Empty column had very few sequences in both pools with only 4 in SELEX and 8 in RAPID. In addition, the majority of the Empty-column sequences had enrichment values less than one between the two cycles, which is expected if the binding and copy number for those sequences is random.

### Independent RAPID and SELEX Enrich Identical Sequences

**Figure 5:**
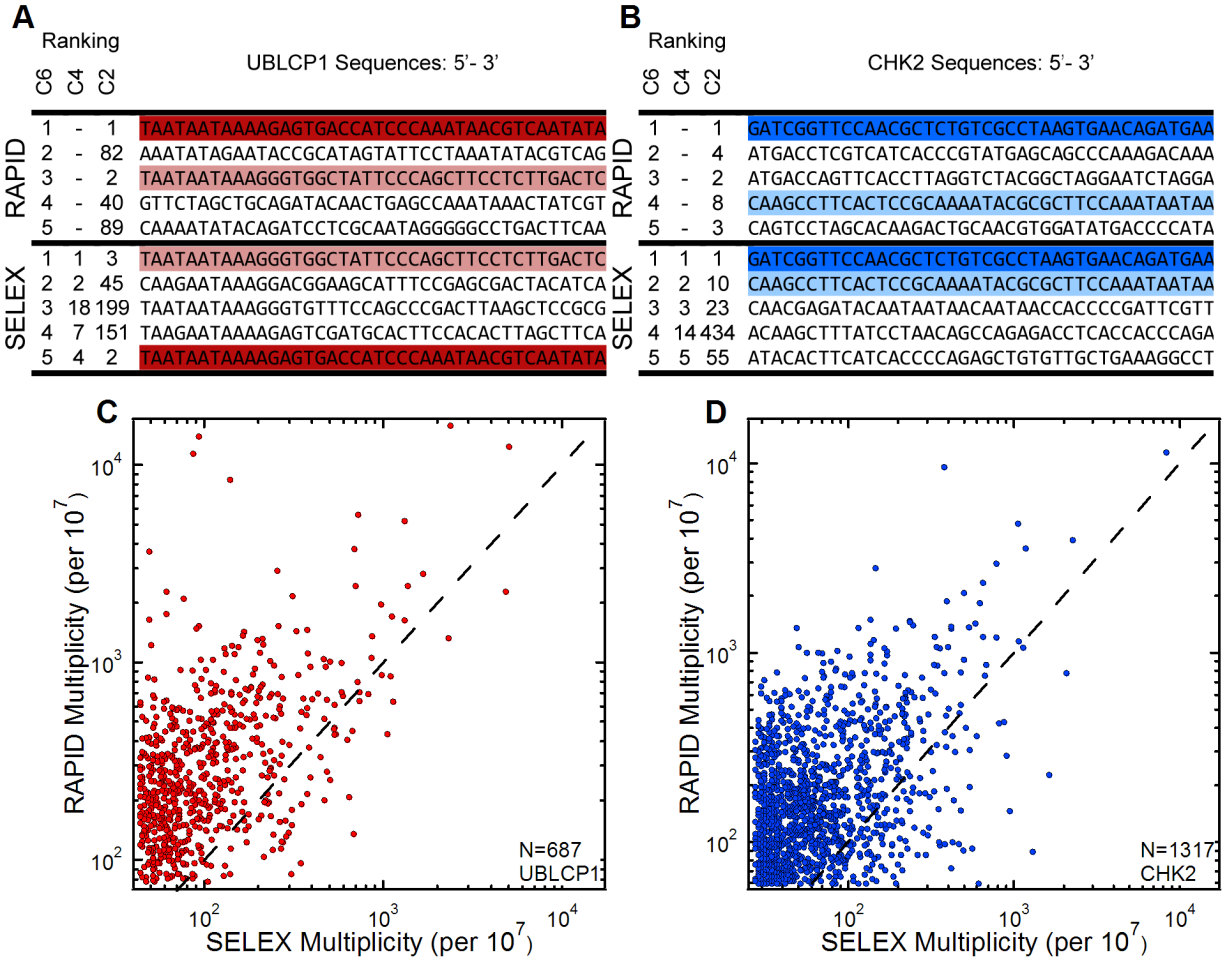
Relationship of the SELEX and RAPID selected sequences in Cycle 6 pools. (A and B) The first 40 random bases of the top 5 UBLCP1 and CHK2 sequences from Cycle 6 in RAPID (top) and SELEX (bottom). Identical sequences between both methods are highlighted with matching colors. The ranks of each sequence at earlier cycles (4, 5 and 6) are also shown. (C) A scatter plot of the 687 common sequences for UBLCP1 in SELEX and RAPID Cycle 6 pools; the dashed line represents a 1∶1 correlation between multiplicities in the two pools. (D) The same analysis for CHK2 yielded 1317 common sequences. On average, RAPID pools were enriched above SELEX pools.

A closer examination of the sequencing results for the two Cycle 6 pools of each protein revealed identical sequences that had achieved very high multiplicities in both RAPID and SELEX. Among the top five candidates, UBLCP1’s highest-ranked sequence in RAPID was ranked fifth in SELEX and its top-ranked sequence in SELEX was ranked third in RAPID (Figure 5A). Furthermore, the top-ranked CHK2 sequence in RAPID was also the top ranked sequence in SELEX (Figure 5B). This analysis was done using the entire random region of each candidate (i.e. not a short sequence motif), so each sequence represented the identical sequence that was selected from the 5×10^15^ random sequence library using RAPID and SELEX.

To extend this analysis, we searched for additional sequences common to each target’s RAPID and SELEX Cycle 6 pools and found that many sequences among their top 10,000 were common and highly represented in both methods. Scatter plots relating the multiplicities of sequences represented in both pools are shown in Figures 5C and 5D. In total, we found 687 sequences that were common in both UBLCP1 pools and 1317 sequences that were common in both CHK2 pools. Analysis for the Empty column yielded only a single common sequence with negligible multiplicities. It is difficult to prove that identical sequences identified in multiple selections are not the result of cross-contamination between simultaneous side-by-side selections; however, RAPID and SELEX were performed independently of each other at different times making contamination between methods unlikely. In addition, almost all of the common sequences were unique to each target (Figure S2) and most appeared more highly enriched in the RAPID Cycle 6 pools. On average, the RAPID selected sequences represented higher fractions of their pools having enriched approximately 3-fold more than from SELEX: UBLCP1 by a factor of 2.6 ⋇ 2.3 (1.1–6.0-fold) and CHK2 by a factor of 2.8 ⋇ 2.2 (1.3–6.2-fold). These were determined by finding the geometric mean and standard deviation for the enrichments, thus the enrichments and their standard deviations are expressed as multiplicative factors.

### Aptamer Binding to CHK2 Protein

**Figure 6:**
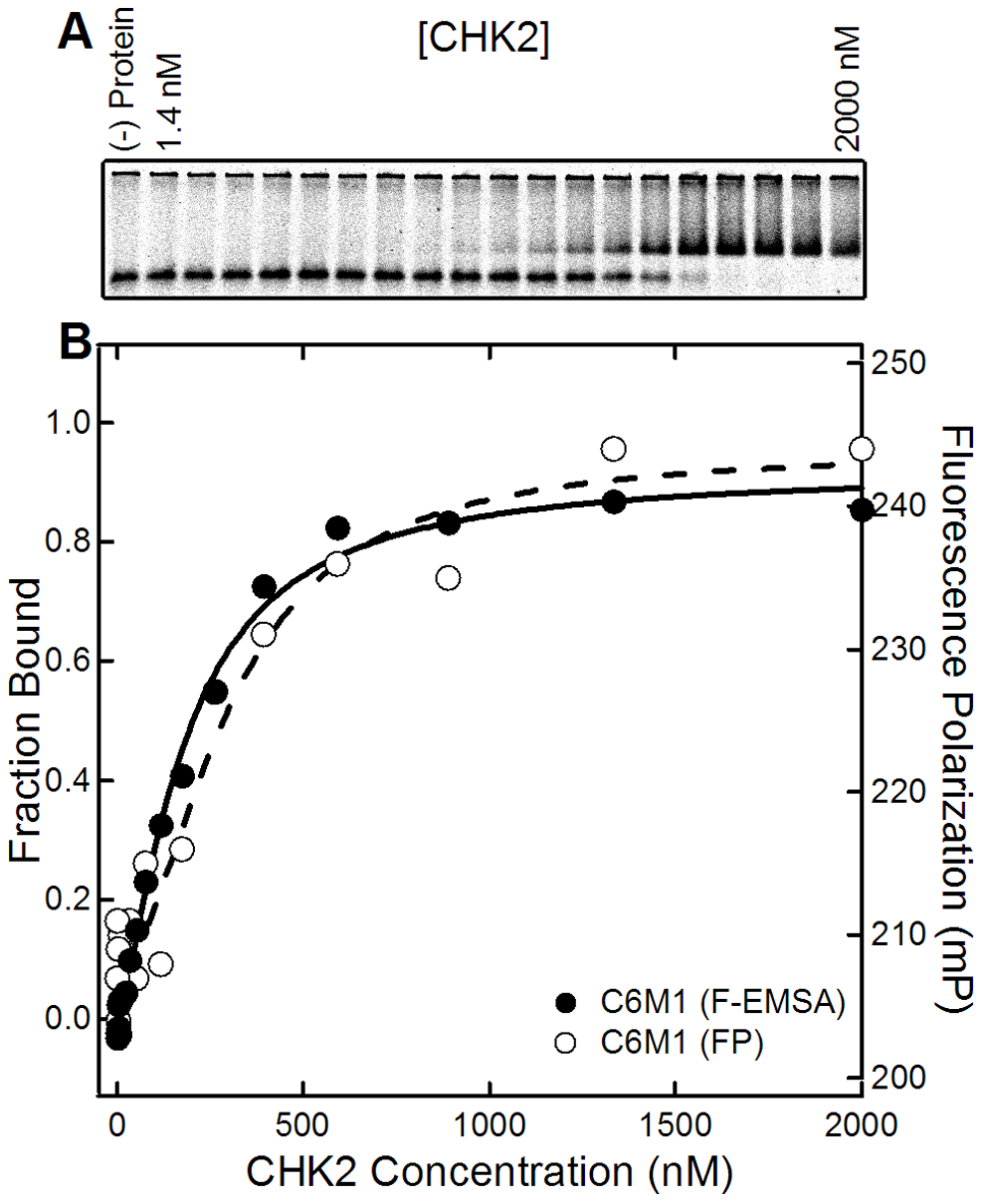
Binding test of the CHK2 protein prep’s highest multiplicity Cycle 6 aptamer candidate C6M1. The sequence is given by the two flanking constant regions, and the random region: GATCGGTTCCAACGCTCTGTCGCCTAAGTGAACAGATGAAGAAAAAATAGCCCAATAAGAGGCAACAATCT. (A) Gel image of F-EMSA for C6M1 aptamer incubated with no protein or the CHK2 protein prep ranging from 1.4 nM to 2000 nM, in 1.5-fold increments. (B) Binding curves for C6M1 using F-EMSA and FP. The left axis shows the calculated fraction bound from F-EMSA (solid line, black circles), while the right axis shows the fluorescence polarization from C6M1 (dotted line, white circles). The fitted K_d_ for the two curves are 180±13 nM and 299±53 nM, respectively.

The sequence for CHK2 identified as the top-ranked one in both selection methods, hereafter referred to as C6M1, was tested for its binding affinity to CHK2. After C6M1 was isolated from the Cycle 6 pools, it was labeled with fluorescein, and then evaluated via the Fluorescent Electrophoretic Mobility Shift Assay (F-EMSA). Figure 6A shows an image of the resulting gel shift assay. The fraction of bound RNA was evaluated from the gel image and plotted as the filled symbols in Figure 6B. The solid line fit to the data was done using the Hill equation which yielded a K_d_ value of 180±13 nM. In order to ensure that the observed binding was not a gel artifact, a Fluorescence Polarization (FP) assay was also performed. The polarization results and curve fit are shown as the open symbols and dashed line in Figure 6B. The calculated K_d_ is 299±53 nM, which is 1.6-fold higher than determined with F-EMSA. This factor is consistent with other FP assays performed on some of the labeled bulk SELEX pools (Figure S3). Currently, we have not ruled out potential aptamer binding to a contaminant in our protein preparation. If this were the case, given the purity of our preparations, the binding affinity of C6M1 would be underestimated by at least an order of magnitude and thus the approximate K_d_ value would be less than 20 nM. However, for the purposes of this manuscript, the results and conclusions of this work remain the same in either case.

## Discussion

RAPID SELEX is capable of isolating aptamers in less time than conventional SELEX. Standard binding assays with the amplified pools clearly revealed cycle-to-cycle affinity enrichment for two protein targets, CHK2 and UBLCP1, using both RAPID and conventional SELEX. Further, higher affinities and total binding to CHK2 were observed for pools from later selection cycles. We found that the two Cycle 6 pools bound with comparable affinity, although the RAPID pool bound slightly better (∼1.5-fold higher). Even though the RAPID selections were not performed with the optimal conditions used in SELEX, this suggests that the Non-Amplified RAPID pools did not suffer in performance compared to the SELEX pools, which would support the use of RAPID in many if not most selection strategies.

As with the binding affinities, we found that despite having half the amplification steps as SELEX, the RAPID pools had slightly more converged sequence distributions. This is in good agreement with the ordered binding curves mentioned above, which suggested that the RAPID pools should have slightly more converged distributions. This is in fact what we observed (Figure 3 and 4), and recalling our definition of a selection “round” that necessarily includes amplification steps, we found that one RAPID round was most similar to three SELEX rounds in terms of convergence (Figure S1). Similarly, two RAPID rounds yielded convergence similar to five SELEX rounds. This is particularly noteworthy since we found that our top candidate aptamers had acquired their high rankings after just two rounds of RAPID (four cycles).

Finally, we found that among the top 10,000 ranked Cycle 6 sequences from both selections, a large percentage (7% and 13%) were identical. This kind of reproducibility from different SELEX experiments has been addressed before; however, in this past study, sequencing was done at much less depth (less than 100 clones) and the identified aptamers generally contained short motifs which were determined to be highly represented in starting pools [36]. We found no sequence motifs in any of our pools and therefore restricted our analysis to the entire sequence of the ∼70 nt random region. Independent enrichment of the identical rare sequences (∼1 in 10^15^) in both selection methods demonstrate the effectiveness and the robustness of our selection protocols. However, in further support of RAPID, we found that among those identical sequences, the great majority were more enriched an average of ∼3-fold, in the RAPID Cycle 6 pools over the SELEX pools. As mentioned previously, the top aptamer candidates were actually resolved by Cycle 4 in both selections. This reflects the power of high-throughput sequencing for identifying enriching aptamers with great sensitivity many cycles before complete convergence. From these data, we chose to isolate our best candidate aptamer for CHK2, C6M1, and showed that the raw aptamer was indeed able to bind to its target. Further development and characterization of CHK2 and UBLCP1 specific aptamers is beyond the scope of this work and therefore not fully investigated. However, RAPID was able to generate the same results as SELEX in only one third the time.

In addition to specific protein binding results, we studied the impact that the empty microcolumns and downstream processing had on the selections. Interestingly, we noticed that the empty microcolumns generally bound a comparable amount of RNA as the two protein targets (Figure 2). This is not surprising because aptamers are assumed to be rare in the starting library; nearly all the recovered sequences in an initial selection represent background and non-specific binding sequences. Despite this, there was negligible sequence convergence from cycle to cycle (Figure 3). The collective set of high-throughput sequencing results for the Empty microcolumns also suggest that there was negligible sequence bias in the starting library [17] as well as negligible contributions from the microcolumns and the enzymatic processes (PCR, transcription, etc.) to the overall sequence enrichment in the two protein target pools [24].

While we demonstrated RAPID using the simple pairing of one Non-Amplification Cycle followed by one Amplification Cycle, the efficiency of RAPID may be further improved. In general, more Non-Amplification Cycles can be performed between Amplification Cycles, though the number will be limited by practical considerations. Non-Amplification Cycles have the potential to significantly increase the efficiency of selections through the rapid accumulation of affinity enrichments in a short period of time. However, despite higher binding efficiencies, this process also depletes the population of high affinity sequences. Assuming (or requiring) a minimum binding probability, *P_A_,* for a population of aptamers, the number of Non-Amplification cycles can be increased as long as an acceptable copy number of high affinity aptamers, *N_min_,* is estimated to always be present before each cycle *(N_min_* should be chosen such that *N_min_ ≥ (P_A_)^−1^* so that at least one copy of an aptamer is expected to remain after the last cycle). This can be expressed as:
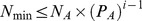
(1)where *N_A_* is the initial (or amplified pool’s) copy number of the aptamer population and *i−1* is the maximum number of Non-Amplification Cycles, with the *i^th^* cycle being an Amplification Cycle which must be done to replenish the pool’s sequence populations. In addition, each Non-Amplification Cycle decreases the input material for the subsequent cycle which may result in increased binding fractions and reduced enrichment yields, diminishing the practicality of continued Non-Amplification Cycles. Using a simple measurement of total binding, the number of Non-Amplification Cycles can be increased as long as an acceptable enrichment, *E_min,_* of high affinity aptamers is estimated to have resulted after each cycle. This can be expressed as:
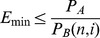
(2)where *P_B_(n,i)* is the background binding probability at the *n^th^* total selection cycle with i cycles since the last amplification. If this expression ever proves false, amplification of the pool can be used to increase the concentration and selection stringency to improve future enrichments.

Together, the above two expressions place upper limits on the total number of Non-Amplification cycles that can be performed between Amplification Cycles, and maximizes the potential efficiency of RNA selections. Applying these expressions to our simple RAPID protocol required a minimum binding probability for aptamer candidates of about 40% (to ensure 1 copy survives the first round) which is typical of binding efficiencies demonstrated on our microcolumns [30]. Taking into account the amount of amplification and the measured background binding over the six cycles, our highest candidates should represent between 1 in 100–1000 sequences. In fact our top candidates are represented in the middle of this range. Altogether, our results make a compelling case for RAPID both in its efficiency, and its cycle-to-cycle performance.

Although we used our microcolumn-based processes to perform all selections, RAPID may be used in combination with any selection mode or technology to save time, reagents, and to rapidly converge selected pools. RAPID could be particularly useful for slow selections requiring many cycles, or when complete sequence convergence is needed so that conventional cloning methods can be used to identify candidates. Although the time-saving benefits would be less compared to RNA-based selections, RAPID can also be extended to DNA selections. We used high-throughput sequencing to quantify selected pools as described by histograms of converging multiplicities, and scatter plots of sequence enrichments and identical sequences derived from two independent selection methods. Similar detailed analyses could be used to gain higher confidence in aptamer candidates through replicate selections, or to make more quantitative evaluations of different selection schemes and technologies. In particular, with a standardized pool and target, these analyses could be used to objectively rank, compare, and optimize different selection techniques.

## Materials and Methods

### Protein Preparation

As previously described [30], recombinant hexahistidine-tagged CHK2 and UBLCP1 proteins were expressed in BL21(DE3)-RIPL E. coli cells (Agilent Technologies). LB cultures supplemented with 100 µg/ml ampicillin were inoculated with starter LB culture derived from a single colony and grown at 37°C until OD_600_ reached 0.6. Protein expression was induced with 0.2 mM IPTG at 18–22°C for ∼16 hours. After centrifugation, the bacterial pellet was collected and processed according to the manufacturer’s instructions for Ni-NTA Superflow resin (Qiagen). SDS-PAGE was used to determine the purity and quality of the final protein product. The resulting proteins were dialyzed with 1×PBS with 5 mM 2-mercaptoethanol and 0.01% Triton X-100. The proteins were evaluated for purity (∼90–95%) and were stored in small aliquots with 20% glycerol.

### RNA Library Preparation

As previously described [30], a synthesized DNA library was purchased from GenScript. To increase the diversity of the initial library and to include higher order RNA structural classes, we chose to use a random region of 70 nucleotides (nt); this length averages about 4.5 structural features (vertexes) [37]. Including flanking constant regions, sequences in the library have 120 nts, as described by the scheme: 5′-AAGCTTCGTCAAGTCTGCAGTGAA-N70-GAATTCGTAGATGTGGATCCATTCCC-3′. This length is the practical limit for efficient commercial synthesis of DNA templates. The single-stranded DNA template library was converted to double-stranded DNA while introducing the T7 promoter using Klenow exo- (NEB) and the Lib-FOR oligonucleotide, 5′-GATAATACGACTCACTATAGGGAATGGATCCACATCTACGA-3′. The resulting library was later amplified in a 1 L PCR reaction using Taq DNA polymerase, Lib-FOR oligonucleotide, and the Lib-REV oligo, 5′-AAGCTTCGTCAAGTCTGCAGTGAA-3′. A single aliquot capturing the complexity of the entire library (5×10^15^ unique sequences) was transcribed with T7 RNA polymerase in an 88 mL reaction yielding 1200-fold amplification. An aliquot of this RNA library, corresponding to an average of 4 to 6 copies of each unique sequence, was used as the starting pool for each selection method.

### Multiplex SELEX and RAPID

The protein immobilization was described previously [30]. Briefly, a new batch of resin was prepared for each protein target. Ni-NTA Superflow resin was incubated in binding buffer (25 mM Tris-HCl, pH 8.0, 10 mM NaCl, 25 mM KCl, 5 mM MgCl_2_) with each protein to the optimal final concentration of ∼0.6 µg protein/µl of resin and then loaded into custom fabricated microcolumns [30]. For both SELEX and RAPID, three microcolumns were serially connected beginning with an Empty microcolumn, followed by UBLCP1 and ending with CHK2. Fresh aliquots of the RNA Library were prepared in 1 mL binding buffer by heat denaturing at 65°C for 5 minutes, renaturing at 25°C for 30 minutes and finally adding 200 U of Superase-In RNase Inhibitor (Invitrogen). 10 µL samples were taken as 1% standards for subsequent quantitation by qPCR.

For the SELEX cycles, 1 mL of blocking buffer (binding buffer supplemented with 0.3 µg/µL yeast tRNA) was injected into the microcolumn assembly at a rate of 100 µL/min. The library was injected at the optimum rate of 1 µL/min using a multi-rack syringe pump (Harvard Apparatus) [30]. After binding the library, the microcolumns were reconfigured to run in parallel, and a 3 mL washing step was performed at the optimum rate of 3 mL/min with binding buffer containing 10 mM imidazole. Finally, the protein and bound sequences were collected from the microcolumns by flowing 400 µL of elution buffer (binding buffer supplemented with 50 mM EDTA) at 50 µL/min. By chelating the nickel ion (Ni^+2^) from the resin with EDTA, protein-resin binding was disrupted allowing the recovery of all protein-RNA complexes and thus avoiding elution bias against potential Mg-independent binding aptamers. Each RNA sample was then phenol:chloroform and chloroform extracted, ethanol precipitated together with 1 µL of GlycoBlue (Ambion) and 40 µg of yeast tRNA (Invitrogen), and re-suspended in 20 µL of DEPC-treated water. These were then reverse transcribed, PCR amplified, and transcribed into RNA (see below for details) for the next selection cycle. Five more SELEX cycles using the three microcolumns were completed in parallel, decreasing the washing flow rate by 10-fold at Cycles 3 and 6 to accommodate possible increases in the bulk affinity of the enriched pools. The input material was also decreased by 20-fold each cycle from Cycle 2 to 4 to decrease the time and reagents needed.

For the RAPID cycles, 1 mL of blocking buffer was injected into the serial microcolumn assembly at 100 µL/min. The library injections were performed at 10 µL/min to allow the completion of multiple selection cycles in one day. For the wash step, we used a 3 mL two-step wash at 1 mL/min for 1 minute, followed by 70 µL/min for 29 minutes. This combined the observed benefits of a brief, harsh wash for eliminating weakly bound or unbound molecules, with that of a longer wash for discriminating among more strongly bound molecules [30]. Elution buffer was then injected to recover bound sequences, which were then phenol:chloroform and chloroform extracted, ethanol precipitated, re-suspended in 1 mL binding buffer, and then used as the input pool for the next selection cycle. We took 1% standards/samples from each new pool and then the selection steps were repeated with all of the microcolumns in parallel. Following the completion of the elution step after the second cycle, each RNA sample was extracted, precipitated, and re-suspended in 20 µL of DEPC-treated water and processed for the next selection cycle. Two more RAPID “dual-cycles” (one Non-Amplification and one Amplification Cycle) were completed using the three microcolumns in parallel, decreasing the input material by 20-fold after each amplification cycle (Cycle 3 and 5).

The amplification and quantification of both the SELEX and RAPID pools were performed in the same way. All the resuspended samples and standards were reverse transcribed in 60 µL reactions with MMLV-RT and 30 pmol of Lib-REV primer. The cDNA samples were treated with RNaseH (Ambion) and a small amount analyzed on a LightCycler 480 qPCR instrument (Roche) to determine the amount of RNA that was recovered and to determine the optimal number of PCR cycles. 400 µL PCR reactions with 300 pmol of each primer were performed for each pool, followed by phenol:chloroform and chloroform extractions, and finally purified using DNA Clean & Concentrator (Zymo Research) spin columns. A small fraction (∼1/4) of the purified PCR product was used to generate new RNA pools in 72 µL transcription reactions with T7 RNA polymerase. The template DNA was removed by DNaseI digestion and the resulting RNA pool was purified by phenol:chloroform and chloroform extractions and ethanol precipitation.

### High-throughput Sequencing and Analysis

A detailed description has been reported [30]. Briefly, PCR products from each target pool for various selection rounds were PCR amplified using 6 nt barcoded primers with adapters for the HiSeq 2000 (Illumina) sequencing platform. The barcoded PCR products were PAGE-purified, phenol:chloroform and chloroform extracted, ethanol precipitated, and then re-suspended in 10 mM Tris-HCl pH 7.5 buffer. High-throughput sequencing was performed by the sequencing core facility at Life Sciences Core Laboratories Center, Cornell University. After removing ambiguous and poor scoring sequences the remaining sequences were separated into pools based on the barcode sequences. Then sequences with 85% sequence identity were clustered together. This identity threshold is set to ensure that truly unique sequences with 85% identity (or higher) are unlikely to be present even within our large library size (2.5×10^15^) due to the vast potential 70 nt random sequence space (4^70^ = ∼1.4 10^42^) and thus such detected sequences account for PCR and sequencing errors. The sequence with the highest number of reads, hereafter referred to as the sequence multiplicity, within each cluster was identified as the cluster’s true sequence and used as the representative sequence for that cluster. The total multiplicity of a cluster was defined as the sum of multiplicities within the cluster. All the representative sequences in each pool were sorted based on their multiplicity to identify candidate aptamers for each protein target. The top 10,000 highest multiplicity sequences for each pool are provided in Supporting Information S1. Sequence comparisons, histograms and scatterplots were performed and generated in MATLAB (Mathworks).

### Candidate Sequence Purification

The DNA templates for candidate aptamers were PCR amplified from the final Cycle 6 pool using Phusion Polymerase (NEB), the Lib-REV oligonucleotide, and an aptamer-specific oligonucleotide that spans the forward constant region and approximately 30 nt of the candidate’s unique, random region. The resulting PCR product was double-digested with BamHI and PstI, and ligated using low melt agarose “in-gel” ligation (EZ Clone Systems) into a similarly cut pGEM3Z-N70Apt plasmid. PGEM3Z-N70Apt plasmid was obtained by cloning a random full-length aptamer template from the N70 library together with T7 promoter into the pGEM3Z vector (Promega) between NarI and HindIII sites. Three clones were sequenced to obtain a consensus for the full-length sequence of each candidate aptamer. The RNA aptamer was transcribed from the candidate’s DNA templates, which were generated by PCR from the sequenced plasmid using the same primers.

### Fluorescence EMSA and Polarization Assays

The RNA samples were 3′-end labelled with fluorescein 5-thiosemicarbazide (Invitrogen) as described previously [38]. 50 µL binding reactions were prepared with 2 nM fluorescently-labelled RNA and decreasing amounts of protein (2000 to 0 nM) in binding buffer containing 0.01% IGEPAL CA630, 10 µg/ml yeast tRNA, and 3 U of SUPERase•In RNase Inhibitor. Reactions were prepared in black 96-well half area microplates (Corning) and incubated at room temperature for 2 hours. The plates were scanned on a Synergy H1 microplate reader (BioTek) using the Ex: 485/20 Em: 528/20 filter set to determine the Fluorescence Polarization (FP). The polarization *P* is determined from the total parallel and perpendicular polarized fluorescence according to:
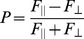
(3)


For Fluorescence Electrophoretic Mobility Shift Assays (F-EMSA), the same samples used for the FP measurements were spiked with 6× loading dye and loaded into the wells of a refrigerated 5% agarose gel prepared with 0.5×TBE buffer. The gel was run for 90 minutes at 120 volts in refrigerated 0.5×TBE buffer. Images were acquired using the fluorescein scan settings on a Typhoon 9400 imager (GE Healthcare Life Sciences) and the resulting bands were quantified with ImageJ. The dissociation constant, K_d_, was determined by fitting the binding results, *Y*, from the FP and F-EMSA to the Hill equation:
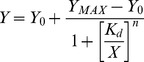
(4)where Y*_MAX_* is the maximum signal from binding, Y*_0_* is background, *n* is the Hill coefficient, and *X* is the protein concentration.

## Supporting Information

Figure S1 **The similarity between RAPID and SELEX pool distributions.** For each target, similarity between pools is determined by calculating the percent overlap of each RAPID cycle’s distribution with each SELEX cycle’s. The highest valued SELEX cycle against a given RAPID cycle is considered to be most similar to the given RAPID cycle. For both protein targets, the RAPID pools Cycle 2 and 4 distributions are most similar to the “later” SELEX Cycle 3 and 5 distributions, respectively. For the Empty columns, the overlap values are close to 100% between all of the pools confirming that there was negligible sequence convergence beyond the initial library within the Empty column’s pools.
(TIF)

Figure S2 **Sequences that are common to both UBLCP1 and CHK2 selected RAPID Cycle 6 pools.** Of the 2004 sequences of interest (687 and 1317 sequences common between Cycle 6 of RAPID and SELEX pools for UBLCP1 and CHK2, respectively), only 8 of them were also common between the two target pools. This is likely due to a trace cross-contamination and strongly suggests that the unique sequences in each pool are target specific.
(TIF)

Figure S3 **Fluorescent polarization binding assays of bulk SELEX pools to CHK2.** The fitted Kd’s for the Cycle 3 and Cycle 6 pools are higher than F-EMSA (Fig. 2). All of the tested pools and C6M1 have calculated dissociation constants 1.6-fold higher when measured from fluorescence polarization compared to F-EMSA.
(TIF)

Supporting Information S1 **Top 10,000 sequences for all pools.**
(XLSX)
