# Supplementary figures and images for: RAPID-SELEX for RNA Aptamers
Source: PLoS One. 2013 Dec 20;8(12):e82667. doi: 10.1371/journal.pone.0082667 (PMC3869713; doi:10.1371/journal.pone.0082667)

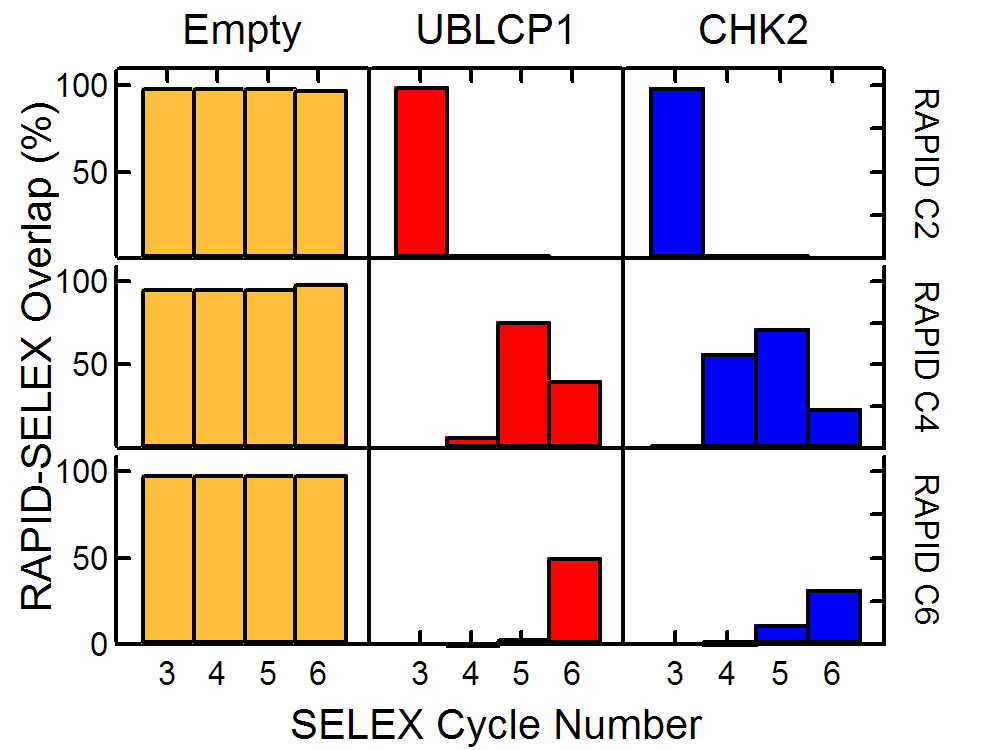

Supplement: Figure S1 — The similarity between RAPID and SELEX pool distributions. For each target, similarity between pools is determined by calculating the percent overlap of each RAPID cycle’s distribution with each SELEX cycle’s. The highest valued SELEX cycle against a given RAPID cycle is considered to be most similar to the given RAPID cycle. For both protein targets, the RAPID pools Cycle 2 and 4 distributions are most similar to the “later” SELEX Cycle 3 and 5 distributions, respectively. For the Empty columns, the overlap values are close to 100% between all of the pools confirming that there was negligible sequence convergence beyond the initial library within the Empty column’s pools. (TIF) [file pone.0082667.s001.tif]

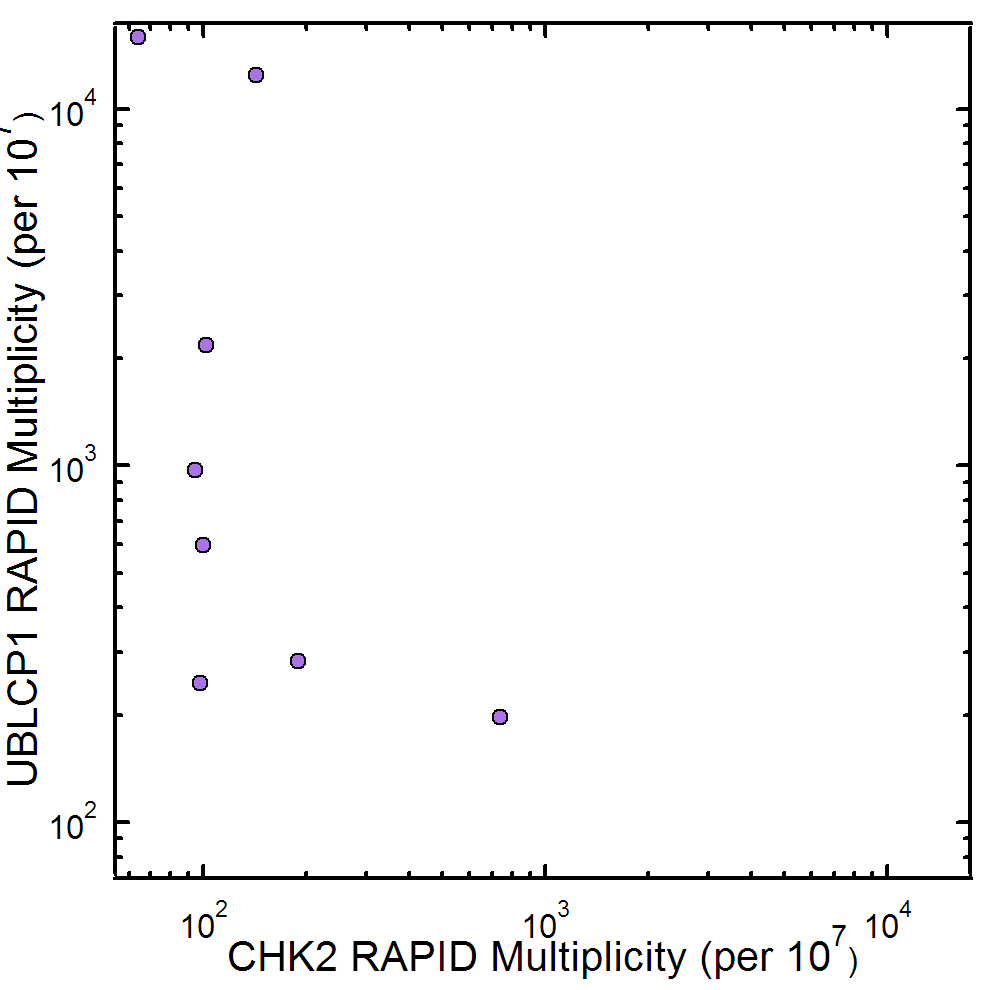

Supplement: Figure S2 — Sequences that are common to both UBLCP1 and CHK2 selected RAPID Cycle 6 pools. Of the 2004 sequences of interest (687 and 1317 sequences common between Cycle 6 of RAPID and SELEX pools for UBLCP1 and CHK2, respectively), only 8 of them were also common between the two target pools. This is likely due to a trace cross-contamination and strongly suggests that the unique sequences in each pool are target specific. (TIF) [file pone.0082667.s002.tif]

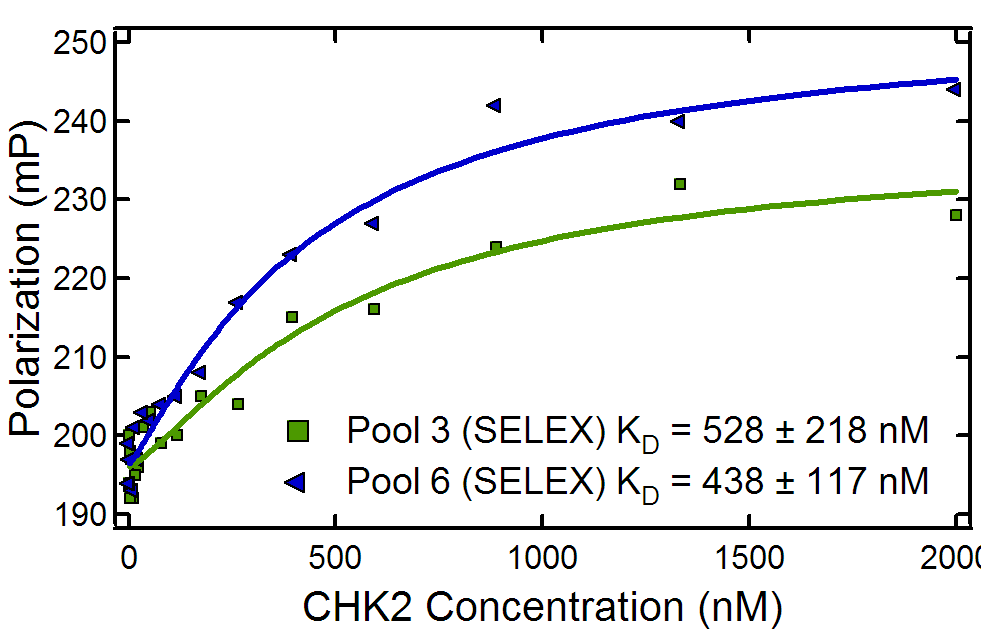

Supplement: Figure S3 — Fluorescent polarization binding assays of bulk SELEX pools to CHK2. The fitted Kd’s for the Cycle 3 and Cycle 6 pools are higher than F-EMSA (Fig. 2). All of the tested pools and C6M1 have calculated dissociation constants 1.6-fold higher when measured from fluorescence polarization compared to F-EMSA. (TIF) [file pone.0082667.s003.tif]
